# Comment - Louis XIV’s terminal illness clinical chronology favors necrotizing soft-tissue infection

**DOI:** 10.1186/s12882-026-05328-8

**Published:** 2026-09-02

**Authors:** Alexis Demas

**Affiliations:** ARGOS (Arts, Research and Gestures Observed Scientifically) Neurosciences and Arts, 229 rue Saint-Honoré, Paris, 75001 France

## Abstract

Louis XIV’s rapidly progressive terminal illness may be better explained by necrotizing soft-tissue infection than primary calciphylaxis. Severe distal pain, ascending gangrene, and fatal sepsis within three weeks suggest fulminant infection, plausibly arising from a diabetic foot lesion. Clinical chronology should remain central when evaluating competing retrospective clinicopathological hypotheses.

**Dear Editor**,

I read with great interest the article by Bataille and Charlier, which proposes chronic kidney disease complicated by calciphylaxis as a plausible framework for Louis XIV’s terminal illness, while explicitly acknowledging that retrospective confirmation is impossible [1]. Their reappraisal illustrates the enduring value of applying modern medical concepts to historical disease. I write not to contest their hypothesis but to place a second one beside it, and to propose the criterion by which readers might weigh the two.

Clinical diagnosis begins with disease tempo. Before considering uncommon entities, physicians first ask which process best explains how an illness evolved. The primary question is therefore not only which chronic conditions the King may have harboured, but which mechanism produced the rapidly progressive syndrome that ended his life.

The historical descriptions are consistent. In early August 1715 the King developed sudden, excruciating pain in the left foot. Within days, tissue discoloration appeared and extended proximally to the leg and thigh before culminating in systemic deterioration and death less than three weeks later. Disproportionate pain preceding extensive necrosis, rapid contiguous extension, and fatal septic collapse constitute the classical presentation of necrotizing soft-tissue infection (NSTI) [2].

It is on this chronology that the two hypotheses may be compared. Calciphylaxis is well recognised to be complicated by secondary infection, and such a sequence cannot be excluded. Less readily accounted for is that primary calcific arteriolopathy is not typically characterised by rapidly ascending fascial destruction culminating in fulminant septic shock within three weeks.

Nothing here requires the King’s kidneys to have been healthy. Long-standing diabetes and obesity make chronic kidney disease entirely plausible, and it may well have coexisted with the mechanism proposed below. The difference between the two readings concerns not what the King carried, but what drove the final three weeks.

A second interpretation emerges from the same metabolic background. Historical observations have long suggested advanced diabetes mellitus, a condition associated with chronic plantar ulceration, foot infection, and fulminant lower-limb sepsis [3]. In the pre-antibiotic era, a neuropathic plantar lesion would have constituted an ideal portal of entry for anaerobic bacteria. The illness then forms a single continuum: chronic ulceration, bacterial inoculation, rapidly progressive NSTI, myonecrosis, septic shock, and death [4]. 

No contemporaneous account documents such an ulcer. The portal of entry is inferred, exactly as chronic kidney disease is inferred in the alternative reading. Both hypotheses rest on an undocumented antecedent and differ in what they require it to explain.

Among potential pathogens, *Clostridium perfringens* is instructive rather than a specific attribution: abrupt severe pain, toxin-mediated muscle necrosis, rapid proximal extension, profound toxicity, and very high untreated mortality [5]. No historical source permits a microbiological diagnosis, and other anaerobic or polymicrobial necrotizing infections would fit comparably.

The appeal of this reconstruction is its parsimony: distal onset, disproportionate pain, contiguous extension, diffuse gangrene, and terminal septic deterioration all arise from a single well-established process.

Historical clinicopathological diagnosis should be governed by the principles that guide bedside medicine: hypotheses should emerge from the documented chronology rather than requiring the chronology to fit a preferred model. Neither hypothesis can be confirmed, and both are advanced in that spirit. I suggest only that necrotizing soft-tissue infection, plausibly complicating a chronic diabetic foot lesion, deserves at least equal standing in the differential, and I leave readers to judge which better accommodates the sequence the sources describe.

## Data Availability

No datasets were generated or analysed during the current study.
